# Two new desert *Eschscholzia* (Papaveraceae) from southwestern North America

**DOI:** 10.3897/phytokeys.35.6751

**Published:** 2014-03-11

**Authors:** Shannon M. Still

**Affiliations:** 1 Department of Plant Science and Conservation, Chicago Botanic Garden, 1000 Lake Cook Road, Glencoe, IL 60022

**Keywords:** Arizona, California, *Eschscholzia*, Papaveraceae, new species, *Eschscholzia androuxii*, Joshua Tree poppy, *Eschscholzia papastillii*, cryptic desert poppy, Mojave Desert, Colorado Desert, Sonoran Desert

## Abstract

Two new species of *Eschscholzia* are described. Both are found in the deserts of California and one extends outside the state boundary into Arizona. *Eschscholzia androuxii* Still, **sp. nov.** is found mainly in and around Joshua Tree National Park in Riverside and San Bernardino counties. *Eschscholzia papastillii* Still, **sp. nov.** is found from the northern Mojave south through Joshua Tree National Park to central Imperial County. Both are annuals found in coarse, sandy soil and have yellow flowers typical of desert *Eschscholzia*. *Eschscholzia papastillii* has an expanded receptacular rim similar to that of *Eschscholzia californica*. *Eschscholzia androuxii* has anthocyanin bands around the stamen filaments.

## Introduction

*Eschscholzia* Cham. (1820) is a genus in the Papaveraceae tribe Eschscholtzieae along with the genera *Hunnemannia* Sweet and *Dendromecon* Benth. The type genus is native to the mainland and islands of western North America in both the United States and Mexico, but the type species, *Eschscholzia californica* Cham. has invaded Mediterranean regions around the world. The taxa are native to mesic and xeric landscapes. Recent treatments for *Eschscholzia* (Clark 1997, Roberts 1989) recognize 12 species and several subspecies for a total of 16 taxa. Recent phylogenetic work (Still and Potter 2013) indicates 18 distinct taxa. While the genus is fairly small, there are nearly 198 taxon names and 168 type specimens, with the majority described by EL Greene (1905). The majority of described taxa are synonymous with *Eschscholzia californica*.

All taxa are herbaceous annuals or perennials with taproots and basal rosettes. The leaves are ternately-dissected 2–many times and range from bright green, dark green to glaucous grey-green. Flowers are bisexual, have two sepals fused into a single cap structure, four petals and many stamens. The sepals fall from the flower upon opening. The flowers are yellow, orange or can be yellow with an orange basipetal spot on each petal. The desert taxa of the genus can be difficult to identify (personal experience) and this resulted in further morphological and molecular examination of *Eschscholzia* (Still 2011, Still and Potter 2013). In the course of study, two new taxa were discovered among *Eschscholzia* native to desert regions (Still 2011). These two taxa exhibited both morphological traits and mutations in nucleotide sequences from nuclear and plastid regions that distinguish them from one another and from all previously described species of *Eschscholzia*. The two new taxa are here described as new species.

## Taxonomy

### Eschscholzia androuxii

Still sp. nov.

urn:lsid:ipni.org:names:77136479-1

http://species-id.net/wiki/Eschscholzia_androuxii

Figs 1 [Fig F2] –3

#### Type.

**UNITED STATES**, **California:** Riverside County, just south of entrance sign to Joshua Tree National Park heading north on Cottonwood Springs Road from US Interstate-10, [33°41.188'N, 115°48.103'W], 610 m alt., 14 Feb 2008, *Shannon M. Still 258A with Jennifer Still and Charles Still* (holotype: DAV!).

#### Diagnosis.

*Eschscholzia androuxii* is similar to *Eschscholzia minutiflora* subsp. *twisselmannii* C. Clark & M. Faull but with ultimate lobes of the dissected leaves more numerous and narrower than *Eschscholzia minutiflora* subsp. *twisselmannii*. *Eschscholzia androuxii* is similar to *Eschscholzia minutiflora* subsp. *minutiflora* S. Watson and *Eschscholzia minutiflora* subsp. *covillei* (E. Greene) C. Clark but with larger flowers and consistently appearing, pronounced black-blue or darkened anthocyanin area or spot basipetally located on the fused filament bases of the stamens. *Eschscholzia androuxii* differs from *Eschscholzia papastillii* and *Eschscholzia parishii* with the aforementioned stamen spot and basal foliage that appears more compact in habit.

#### Description.

Annual herb, erect or spreading with a basal rosette of leaves from a taproot. *Leaves* highly ternately-dissected into a great number of ultimate lobes, which may number to 100 on larger specimens. Leaves glaucous-green with ultimate lobes more rounded than pointed. Basal leaves are 3–11 cm long and 0.8–3.2 cm wide and held on a petiole comprising 2/3 the entire leaf length. Younger plants will have fewer ultimate lobes and shorter, narrower leaves. *Inflorescence* with few flowers held above the foliage and to 4 dm above the ground. Leaves on the inflorescence are 2–20 mm long and are divided into 2–23 ultimate lobes. *Buds* nodding and 4.5–11.5 mm long with an apiculate bud tip less than 25% of the total bud length. Less mature buds may be shorter than average with a longer bud tip by percentage. *Flowers* held upright and are yellow with four petals 10.5–23 mm long. Each flower has 20–36 stamens fused at the base. There is a darkened area or patch, often black-blue, located at the fused filament bases of the stamens. Receptacles obconic and 2.5–5.5 mm long and 1.1–3 mm wide and often have a scarious inner hyaline rim. *Fruit* 3.5–6.5 cm long with 10–12 nerves, dehiscing at maturity. Seeds with reticulate ridges.

**Figure 1.**
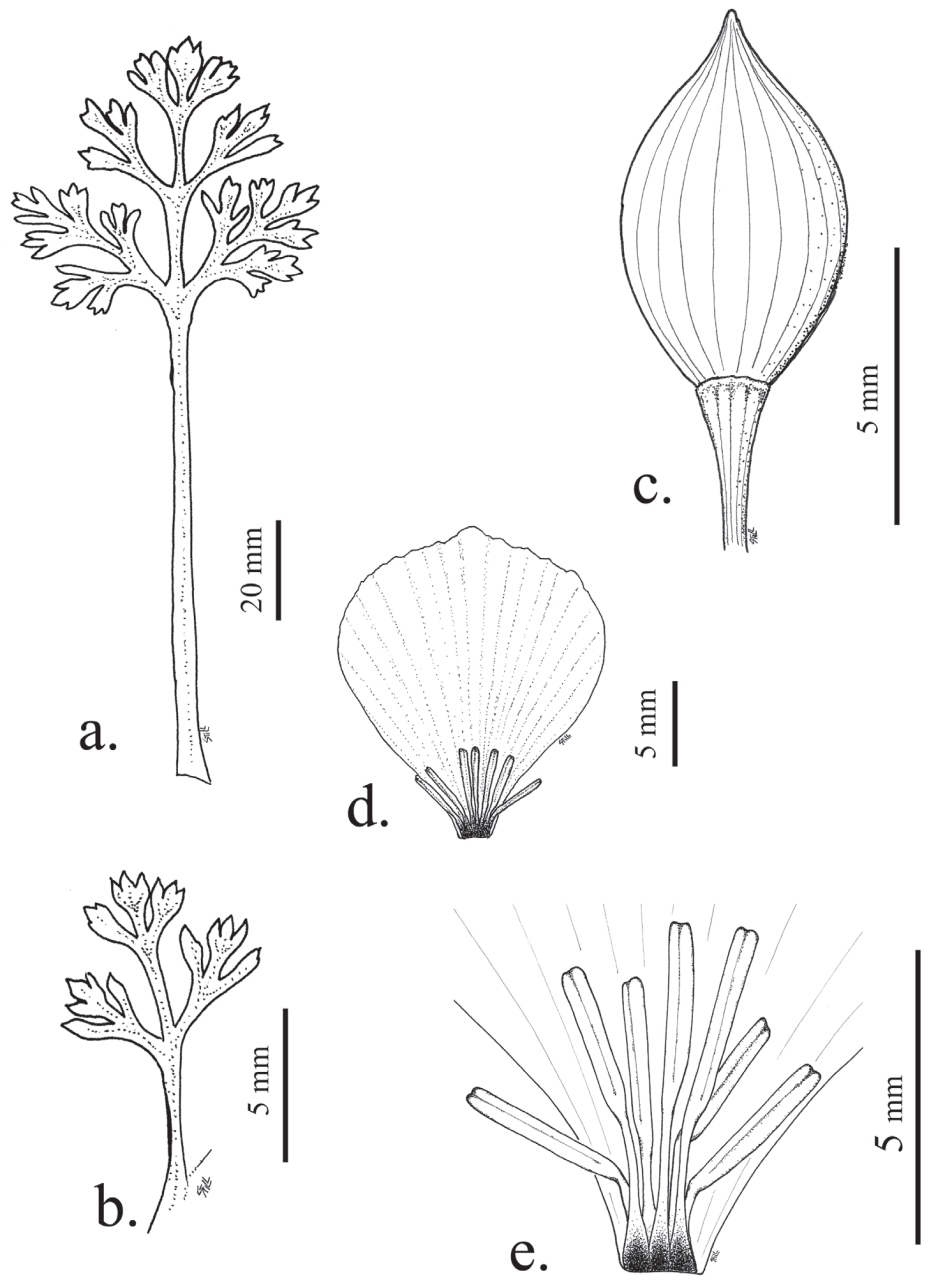
Illustrations of leaves, buds and flowers of *Eschscholzia androuxii*. **A** Basal leaf **B** Cauline leaf **C** Bud **D** Petal **E** Stamens showing diagnostic anthocyanin spot at base of the fused filaments.

**Figure 2. F2:**
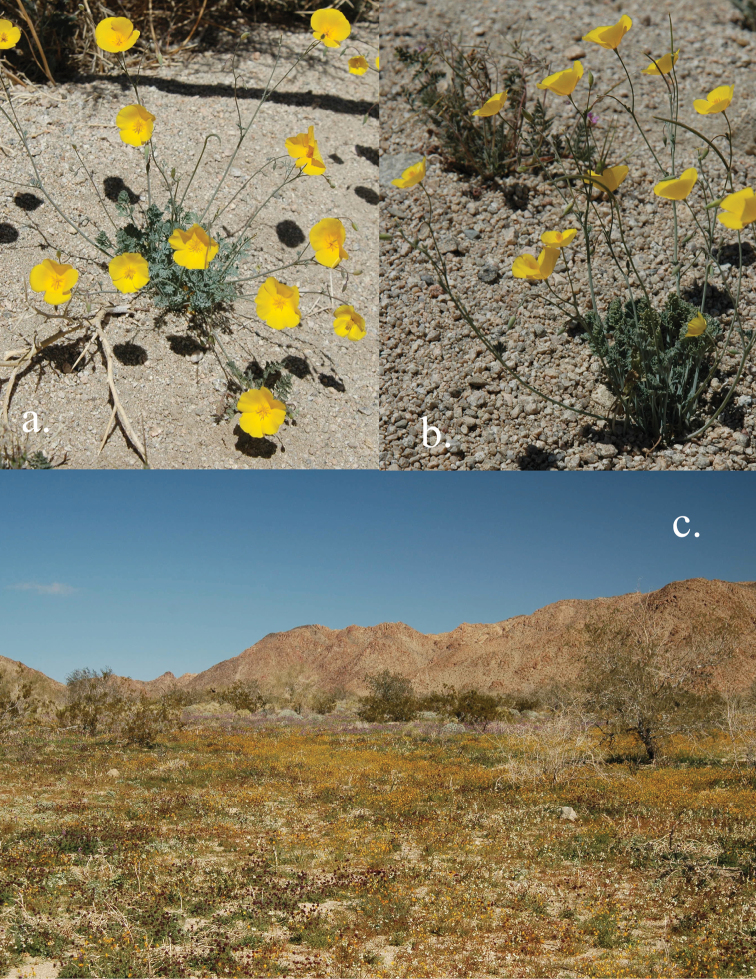
Photographs of *Eschscholzia androuxii*. **A** Species profile shot **B** Species profile shot **C** *Eschscholzia androuxii* in the type area in a heavy-flowering year.

**Figure 3.**
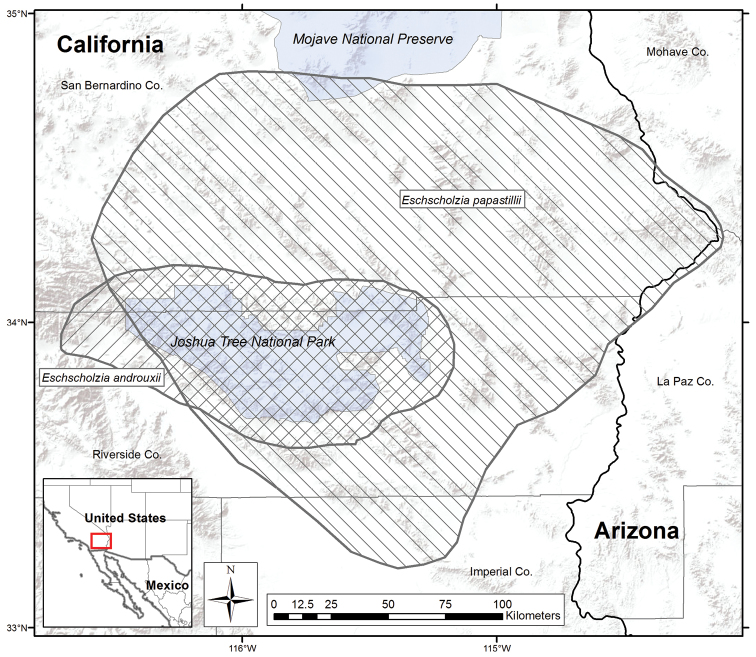
Distribution map showing the range for the two new *Eschscholzia*. *Eschscholzia androuxii* is represented by right-slanting cross-hatching. *Eschscholzia papastillii* is represented by left-slanting cross-hatching.

#### Distribution

(Fig. 3). Found in and around Joshua Tree National Park in both Riverside and San Bernardino counties of California.

#### Habitat and ecology.

Desert washes, flats, and slopes in coarse, sandy soil.

#### Phenology.

*Eschscholzia androuxii* typically flowers between late-February and early-May but may flower earlier in the season, including in the fall, during years with a summer rain and cool fall temperatures.

#### Etymology.

The species is named for James André and Tasha La Doux, two desert botanists and friends that helped point to the problems with desert *Eschscholzia* identification.

#### Suggested common name.

Joshua Tree poppy.

#### Conservation status.

As this is a new taxon it has yet to be considered for conservation status. Due to the limited range and low number of occurrences, the author suggests the California Native Plant Society consider this taxon for listing as a rare plant.

#### Specimens examined.

**U.S.A.** **California:** Riverside Co.: White Water, Apr 1907, *S.B. Parish 6103* (DS!); slope of hill at west side of mouth of Whitewater Canyon, 18 Mar 1962, *D.W. Kyhos 62-43* (DS!); Cottonwood Pass, Joshua Tree National Monument, 19 Mar 1949, *C. Francis Shutts 58* (ASU!, DES!); Coachella Valley, Desert Hot Springs, N of intersection of Pierson Blvd. and Atlantic, north of flood control ditch, 6 Apr 2001, *A.C. Sanders, Mitch Provance & T.B. Salvato 23939* (DES!); Joshua Tree National Park, [33°41.18333'N, 115°48.1'W], 15 Feb 2008, *Shannon M. Still, Jennifer R. Still, & Charles M. Still 258B* (DAV!); id., Cottonwood Wash on west side of road, [33°41.21299'N, 115°48.15100'W], 22 Feb 2009, *Shannon M. Still 444* (DAV!); id., [33°41.833'N, 115°48.17598'W], 3 Mar 2009, *Shannon M. Still & Robert Lee 457* (DAV!); id., [33°50.22799'N, 115°45.174'W], 28 Mar 2009, *Shannon M. Still, Steven M. Still & Carolyn M. Still 512* (DAV!); San Bernardino Co.: 1.4 mi N of Yucca Valley on road to Lucerne Valley. About 19 mi west of town of Twentynine Palms, 6 Apr 1957, *John H. Thomas 6627* (DS!).

#### Discussion.

**Figure 4. F4:**
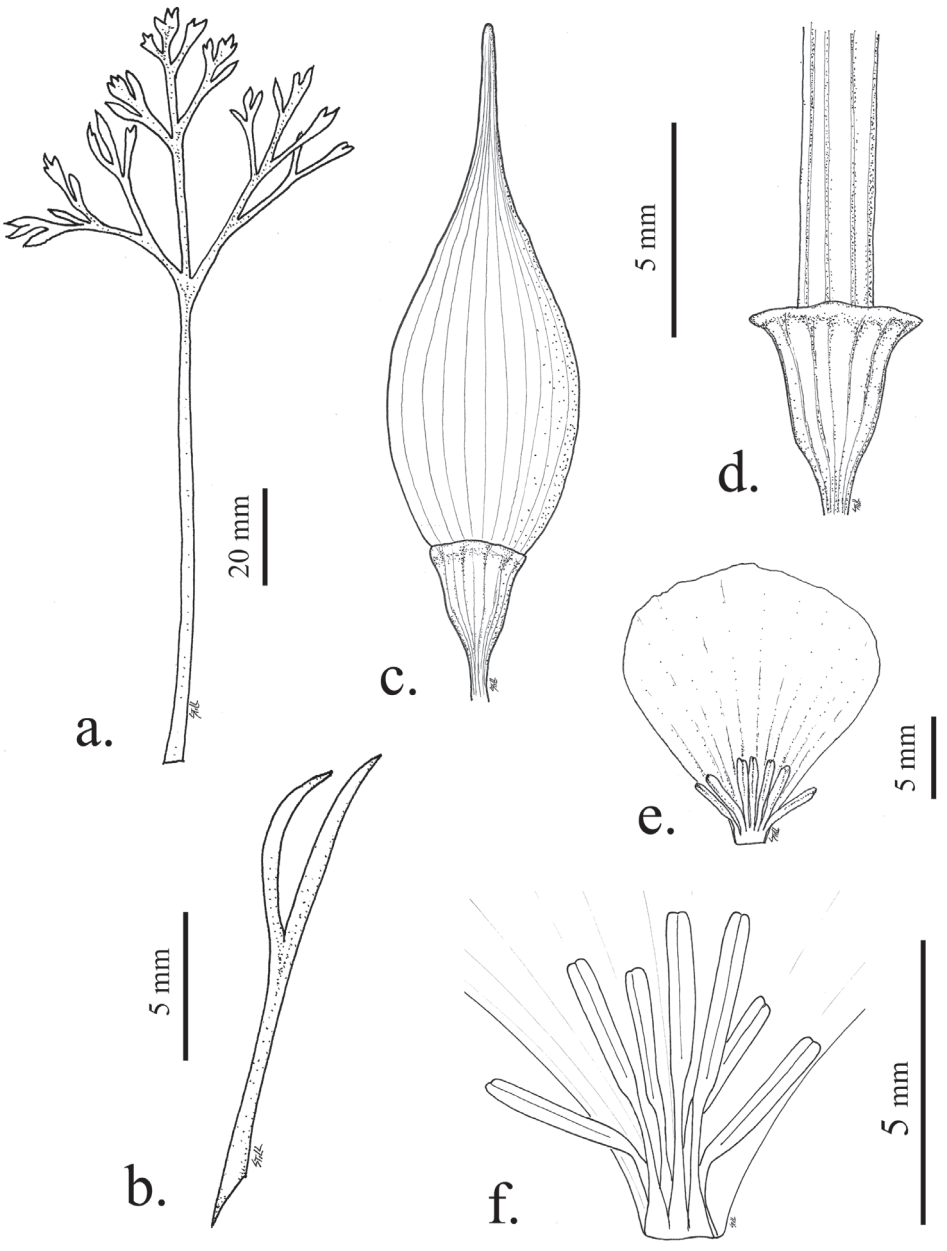
Illustrations of leaves, buds and flowers of *Eschscholzia papastillii*. **A** Basal leaf **B** Cauline leaf **C** Bud showing widened receptacle **D** Enlarged receptacle with expanded receptacular rim common in the species **E** Petal **F** Stamens lacking the anthocyanin spot at base of the fused filaments common to *Eschscholzia androuxii*.

This new taxon has a darkened area basipetally located on the stamen filaments (Fig. 1e), which are fused at the base. *Eschscholzia minutiflora* subsp. *twisselmannii* also has regularly occurring stamen spots, but only on approximately 70% of specimens examined. No other closely related taxa have these stamen spots. The flower size for this new species is similar to that of the diploid *Eschscholzia minutiflora* subsp. *twisselmannii* but larger than both the hexaploid *Eschscholzia minutiflora* subsp. *minutiflora* and the tetraploid *Eschscholzia minutiflora* subsp. *covillei*. The petal size for *Eschscholzia minutiflora* subsp. *covillei*, described in Flora of North America as 6–18 mm long, does overlap with the petal size of *Eschscholzia androuxii*, with petals 10–23 mm long. But more recent morphological study of the genus (Still 2011, Still in preparation) indicates that the petals in *Eschscholzia minutiflora* subsp. *covillei* range from 4.5–12.5 mm. The reason for this discrepancy may be that some of the larger-flowered *Eschscholzia minutiflora* subsp. *covillei* specimens are actually the new taxon, *Eschscholzia androuxii*. The Joshua Tree poppy has an overlapping range with several species but is found only in Riverside Co. and the southern part of San Bernardino Co. and not much further north or south of Joshua Tree National Park. The range does not overlap, and there are more basal leaf ultimate lobes, than with *Eschscholzia minutiflora* subsp. *twisselmannii*. The tips of the basal leaf ultimate lobes are more rounded (Fig. 1a) than what is found in either *Eschscholzia parishii* or *Eschscholzia papastillii* (Fig. 4a), and *Eschscholzia androuxii* has three times the number of cauline leaf ultimate lobes (Fig. 1b) as these two taxa.

### Eschscholzia papastillii

Still sp. nov.

urn:lsid:ipni.org:names:77136480-1

http://species-id.net/wiki/Eschscholzia_papastillii

Figs 3 [Fig F4] –5

#### Type.

**UNITED STATES**, **California:** Riverside County, Joshua Tree National Park next to stone outcropping off Old Dale Road. [33°50.232'N, 115°45.12'W], 724 m alt., 19 Apr 2009, *Shannon M. Still 546A* (holotype: DAV!).

#### Diagnosis.

*Eschscholzia papastillii* is similar to *Eschscholzia parishii* Greene but with more basal leaf ultimate lobes and more broadly spreading leaves. *Eschscholzia papastillii* has an enlarged receptacle (Fig. 4d) that is widely-obconic or bell-shaped, and wider at the midpoint of the receptacle than *Eschscholzia parishii*, *Eschscholzia androuxii* or any of the subspecies of *Eschscholzia minutiflora*, which are usually more obconic or funnel-shaped. The expanded receptacular rim of *Eschscholzia papastillii* is similar, but typically smaller, than the expanded receptacular rim of *Eschscholzia californica*. *Eschscholzia papastillii* differs from *Eschscholzia androuxii* and *Eschscholzia minutiflora* with basal foliage that appears less compact in habit.

#### Description.

Annual herb, erect or spreading with a basal rosette of leaves from a taproot. *Leaves* highly ternately-dissected into 17–70 ultimate lobes with the higher number on larger specimens. Leaves glaucous-green to green with ultimate lobes more pointed than rounded. Basal leaves are 2.7–16 cm long and 0.9–7 cm wide and held on a petiole comprising 2/3 the entire leaf length. Younger plants have few basal leaf ultimate lobes and shorter, narrower leaves. *Inflorescence* with few flowers held above the foliage and to 5 dm above the ground. Leaves on the inflorescence are 3–50 mm long and are divided into 1–13 ultimate lobes. *Buds* nodding to erect and 2.5–16 mm long with an apiculate bud tip greater than 30% of the total bud length. Less mature buds may be shorter than average with a longer bud tip by percentage. *Flowers* held upright and are yellow with four petals 5–24 mm long. Each flower has 12–32 stamens fused at the base. Receptacles widely-obconic or funnel-shaped to nearly bell-shaped, 3–9 mm long and 1.5–4.7 mm wide. Receptacular rim typically noticeable and often thick but can be scarious, expanded laterally up to 1.2 mm from the top of the receptacle. The receptacle often has a scarious inner hyaline rim in addition to the outer rim diagnostic of the species. *Fruit* 4–8 cm long with 10–12 nerves, dehiscing at maturity. Seeds with reticulate ridges.

**Figure 5.**
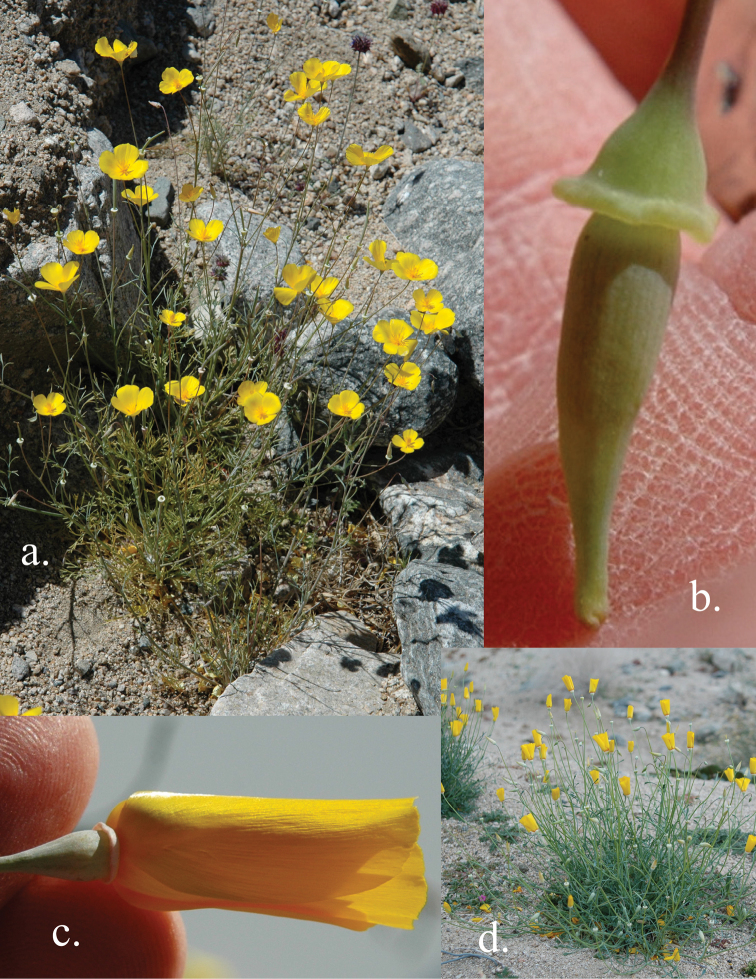
Photographs of *Eschscholzia papastillii*. **A** Species profile shot **B** Buds with enlarged receptacle common in the species **C** Flower with enlarged receptacle common in the species **D** Species profile shot.

#### Distribution

(Fig. 3). Found north to the northern Mojave Desert; south into northern Colorado Desert of San Diego Co., and possibly south along the east side of the Sea of Cortez in Mexico; east to the California-Arizona border (Whipple Mountains); west to the western end of Joshua Tree National Park.

#### Habitat and ecology.

Desert washes, flats, and gentle slopes in coarse, sandy soil.

#### Phenology.

*Eschscholzia papastillii* typically flowers between late-February and early-May but may flower earlier in the season, and in the fall, during years with a summer rain and cool fall temperatures.

#### Etymology.

The species is named in honor of Dr. Steven Still, my father and mentor and the reason for which I study plants.

#### Suggested common name.

Cryptic desert poppy.

#### Conservation status.

As this is a new taxon it has yet to be considered for conservation status. Due to the range and number of occurrences the author does not suggests this taxon be considered for conservation status.

#### Specimens examined.

**U.S.A.** **California:** Kern Co.: Hidden Springs Rd., 6 May 1930, *Lester Rowntree s.n.* (CAS!); Riverside Co.: Painted Canyon, 4 Mar 1922, *Edmund C. Jaeger s.n.* (DS!); Painted Canyon, 12 Apr 1927, *Frank W. Peirson 7167* (CAS!); near Shavers Well, 6 Apr 1930, *R.A. Piebles and H.F. Loomis 188* (DS!); Box Canyon, Coachella Valley, 21 Mar 1937, *Ynez Whilton Winblad s.n.* (CAS!); Coachella Valley, 21 Mar 1937, *Ynez Whilton Winblad s.n.* (CAS!); east slope of Chocolate Mnts, 22 Mar 1937, *Ynez Whilton Winblad s.n.* (CAS!); above Cottonwood Springs, west end of Eagle Mnts, 13 Apr 1949, *Philip A. Munz 13056* (CAS!); Road to Morongo Valley, 7.8 mi from junction with Highway 99/60/70, 14 Apr 1952, *Richard Snow* (DS!); Box Canyon, 5 Apr 1953, *Richard Snow 51a* (DS!); 0.8 mi east of Cactus City, 13 mi east of Coachella, near U.S. Highways 60 and 70. Colorado Desert, 30 Mar 1957, *John H. Thomas 6523A* (DS!); U.S. Highway 60/70, Indio to Blythe, 1 mi. W of Cactus City, 29 Apr 1958, *P.C. Everett and E.K. Balls 23013* (CAS!, DAV!); East of Indio on Highway 60/70, about 6 mi west of Cactus City, 15 Mar 1960, *W.R. Ernst 720* (CAS!); Mecca-Joshua Tree Road, 7 miles southwest of junction with Interstate Highway 10, 13 Apr 1976, *Curtis Clark 527* (DAV!); 2 mi S.E. of Desert Center, 1 Jul 1981, *J.C. Roos s.n.* (ASU!, CAS!); wash along Eagle Mountain Rd., north along I-10, 3 Mar 1995, *John Wear s.n.* (DAV!); Joshua Tree National Park, [33°43.68999'N, 115°49.317'W], 14 Feb 2008, *Shannon M. Still 253 with Jennifer R. Still, Charles M. Still* (DAV!); id., [33°55.012'N, 115°52.60305'W], 3 Mar 2009, *Shannon M. Still 452 with Robert Lee* (DAV!); id., [33°50.22799'N, 115°45.174'W], 28 Mar 2009, *Shannon M. Still 513 with Steven M. Still, Carolyn M. Still* (DAV!); San Bernardino Co.: Sheephole Mnts., 8 Apr 1935, *P.A. Munz 13823* (DS!); Cave Spring, Lower Sonoran zone, 16 Apr 1940, *C.L. Hitchcock 6073* (DS!); Bristol Lake Basin 8.8 mi N. of summit of Sheephole Pass on Amboy Rd., 16 Mar 2001, *A.C. Sanders, Mitch Provance & Petra Wester 23753* (CAS!); Sheephole Pass just to south of the top of the pass, [34°13.711'N, 115°43.19599'W], 25 Nov 2007, *Shannon M. Still 222A* (DAV!); Base of Old Dad Mountains in wash, [34°44.512'N, 115°45.081'W], 26 Nov 2007, *Shannon M. Still, Jim André & Tasha La Doux 248* (DAV!); Base of Old Dad Mountains in wash, [34°44.512'N, 115°45.082'W], 6 Apr 2008, *Shannon M. Still & Steven M. Still 377A* (DAV!); Clipper Mountains, just off the pipeline road, [34°40.573'N, 115°22.73502'W], 18 Apr 2009, *Shannon M. Still, Jim André, Jeff Galvin & Amy Toulsen 536* (DAV!).

#### Discussion.

While the buds of all desert *Eschscholzia* appear similar, those of *Eschscholzia papastillii* most resemble *Eschscholzia parishii*, as the bud tip is typically more than 25% of the total bud length. The receptacular rim is prominent in this species and *Eschscholzia californica* is the only other species that has a pronounced receptacular rim. The range of *Eschscholzia papastillii* extends from San Bernardino County south to northern Imperial County. Most collections of *Eschscholzia parishii* collected north of San Diego and Imperial Counties are likely the new *Eschscholzia papastillii*. *Eschscholzia papastillii* extends at least into easternmost San Bernardino County that contains the Whipple Mountains, and likely well into Arizona.

##### Key to the desert *Eschscholzia* species

**Table:** 

1	Basal leaf ultimate lobes long-linear; leaves ternately-dissected 2–3×; flower scapes typically without cauline leaves; seed coats pitted without reticulations	*Eschscholzia glyptosperma*
1’	Basal leaf ultimate lobes not long-linear; leaves ternately-dissected 3–7×; flowers typically borne on few-flowered racemes with a cauline leaf at each flower axil; seeds coats reticulate
2	Receptacular rim prominent when in fruit, 0.25–5 mm
3	Basal leaf ultimate lobes with length < 3× width, with acute or rounded tips; leaf blades often deep green with a glaucous patch at the crotch of the leaf dissections; cauline leaf ultimate lobes many (range 5–30) with rounded to acute tips; Petals yellow, often with a basipetal orange spot, or petals orange
4	Petals yellow, often with a basipetal orange spot, or petals orange, or rarely white (Arizona mountains); cotyledons entire; annual; limited to eastern Mojave Desert in California and through Arizona	*Eschscholzia californica* subsp. *mexicana*
4’	Petals yellow, often with a basipetal orange spot, or petals orange; cotyledons bifid (2-lobed); annual or perennial; widespread but mostly along highways, railways, and planted areas	*Eschscholzia californica* subsp. *californica*
3’	Basal leaf ultimate lobes with length 3.5 (2–8)× width, with acute tips; leaf blades bright-green to yellow-green; cauline leaf ultimate lobes 3 (rarely 5–13) with acute tips; petals yellow without basipetal orange spot	*Eschscholzia papastillii*
2’	Receptacular rim not prominent in fruit, < 0.25 mm
4	Petal < 1 cm long
5	Buds with tip < 25% total bud length; cauline leaves generally with > 5 (rarely < 6) ultimate lobes, ± rounded to acute; 2n=24 or 36
6	Basal leaf ultimate lobes ± narrow, length ca. 4.5× the width; petals generally less than 5.5 (rarely 2–9) mm long, stamens 6–18, typ. 12; 2n=36	*Eschscholzia minutiflora* subsp. *minutiflora*
6’	Basal leaf ultimate lobes widened, length ca. 2.5× the width; petals generally greater than (5–) 9 (–12) mm long; stamens 6–18, typ. 14–16; 2n=24	*Eschscholzia minutiflora* subsp. *covillei*
5’	Buds with tip > 25% total bud length; cauline leaves generally with ≤ 3 (rarely to 8) ultimate lobes, ± acute to acuminate; 2n=12	*Eschscholzia parishii*
4’	Petals > 1 cm long
8	Bud tip generally > 30% length of bud; leaves bright-green to yellow-green, ultimate lobes ± acute to acuminate; cauline leaf reduced to one-few ultimate lobes
9	Receptacle 1–2 mm wide, obconic to funnel-shaped	*Eschscholzia parishii*
9’	Receptacle 1.5–5 mm wide, widely-obconic to bell-shaped, often flaring at the end of the receptacle	*Eschscholzia papastillii*
8’	Bud tip generally < 20% length of bud; leaves more glaucous to grey-green, ultimate lobes ± round to acute; terminal cauline leaf typically with 5+ ultimate lobes
10	Basal leaves generally with 35–40 (rarely 26–60) ultimate lobes, and ultimate lobes ± widened appearance, length of ultimate lobes less than 2× width, cuneiform; (12–) 18-20 (–28) stamens often with anthocyanin spot at basipetal end of filaments fused at the base; plants of El Paso and Rand Mountains in Kern Co., California	*Eschscholzia minutiflora* subsp. *twisselmannii*
10’	Basal leaves generally with 45–70 (rarely 26–55) ultimate lobes, length of ultimate lobes more than 2× width; stamens (16–) 22-24 (–32), with anthocyanin spot at basipetal end of filaments fused at the base; plants of Riverside and San Bernardino Counties in and around Joshua Tree National Park	*Eschscholzia androuxii*

## Supplementary Material

XML Treatment for Eschscholzia androuxii

XML Treatment for Eschscholzia papastillii

## References

[B1] Chamisso AD (1820) ex Plantis, in expeditione Roman-zonae detectis, genera tria nova. In: CGD Nees von Esenbeck (Ed) Horae Physicae Berolinenses collectae ex symbolis virorum doctorum. Adolphi Marcus, Bonn, 73–74, t. 15.

[B2] Clark C (1997) *Eschscholzia*. In: Flora of North America Editorial Committee (Eds). Flora of North America North of Mexico.New York and Oxford, 3: 308–312

[B3] Greene, EL (1905) Revision of *Eschscholzia*. Pittonia 5: 205-292

[B4] Roberts NC (1989)*Eschscholzia*. In: Baja California plant field guide. Natural History Publishing Company, La Jolla, CA, 234.

[B5] Still SM (2011) Systematic and Taxonomic Studies of Eschscholtzieae (Papaveraceae). PhD thesis, University of California Davis, CA.

[B6] Still SM Potter D (2013) California Poppy Conundrums: Insights into relationships of the tribe Eschscholtzieae (Papaveraceae). Systematic Botany 38: 104-117. doi: 10.1600/036364413X661872

